# Dynamic functional connectivity in verbal cognitive control and word reading

**DOI:** 10.1016/j.neuroimage.2024.120863

**Published:** 2024-09-23

**Authors:** Kazuki Sakakura, Matthew Brennan, Masaki Sonoda, Takumi Mitsuhashi, Aimee F Luat, Neena I Marupudi, Sandeep Sood, Eishi Asano

**Affiliations:** a Department of Pediatrics, Children’s Hospital of Michigan, Detroit Medical Center, Wayne State University, Detroit, MI 48201, United States; b Department of Neurosurgery, Rush University Medical Center, Chicago, IL 60612, United States; c Department of Neurosurgery, University of Tsukuba, Tsukuba 3058575, Japan; d Wayne State University, School of Medicine, Detroit, MI 48202, United States; e Department of Neurosurgery, Yokohama City University, Yokohama 2360004, Japan; f Department of Neurosurgery, Juntendo University, School of Medicine, Tokyo 1138421, Japan; g Department of Neurology, Children’s Hospital of Michigan, Detroit Medical Center, Wayne State University, Detroit, MI 48201, United States; h Department of Pediatrics, Central Michigan University, Mt. Pleasant, MI 48858, United States; i Department of Neurosurgery, Children’s Hospital of Michigan, Detroit Medical Center, Wayne State University, Detroit, MI 48201, United States; j Translational Neuroscience Program, Wayne State University, Detroit, MI 48201, United States

**Keywords:** Pediatric epilepsy surgery, Functional brain mapping, Dynamic tractography, Physiological high-frequency oscillation (HFO), Executive function, Language

## Abstract

Cognitive control processes enable the suppression of automatic behaviors and the initiation of appropriate responses. The Stroop color naming task serves as a benchmark paradigm for understanding the neurobiological model of verbal cognitive control. Previous research indicates a predominant engagement of the prefrontal and premotor cortex during the Stroop task compared to reading. We aim to further this understanding by creating a dynamic atlas of task-preferential modulations of functional connectivity through white matter. Patients undertook word-reading and Stroop tasks during intracranial EEG recording. We quantified task-related high-gamma amplitude modulations at 547 nonepileptic electrode sites, and a mixed model analysis identified regions and timeframes where these amplitudes differed between tasks. We then visualized white matter pathways with task-preferential functional connectivity enhancements at given moments. Word reading, compared to the Stroop task, exhibited enhanced functional connectivity in inter- and intra-hemispheric white matter pathways from the left occipital-temporal region 350–600 ms before response, including the posterior callosal fibers as well as the left vertical occipital, inferior longitudinal, inferior fronto-occipital, and arcuate fasciculi. The Stroop task showed enhanced functional connectivity in the pathways from the left middle-frontal pre-central gyri, involving the left frontal u-fibers and anterior callosal fibers. Automatic word reading largely utilizes the left occipital-temporal cortices and associated white matter tracts. Verbal cognitive control predominantly involves the left middle frontal and precentral gyri and its connected pathways. Our dynamic tractography atlases may serve as a novel resource providing insights into the unique neural dynamics and pathways of automatic reading and verbal cognitive control.

## 1. Introduction

Verbal cognitive control, a fundamental human function, enables focus on relevant information, suppression of automatic reactions, and performance of intricate verbal tasks by extracting critical linguistic details from our environment. This capability is thought to be governed by neural coordination between higher-order cortical regions, such as the prefrontal and premotor cortices, rather than localized neural activity (Brass et al., 2005; Egner and Hirsch, 2005; Fellows and Farah, 2005; Alexander et al., 2007; Gläscher et al., 2012; Oehrn et al., 2014; Jiang et al., 2023). Investigators have reported that lesions in the left prefrontal and premotor white matter regions are correlated with cognitive control deficits, highlighting the critical role of white matter pathways (Mayda et al., 2011; Jiang et al., 2023). Additionally, intraoperative stimulation of the right frontal white matter structures was found to cause transient impairments in verbal cognitive control (Puglisi et al., 2019). While the roles of these cortical and subcortical regions have been suggested, the temporal dynamics of neural coordination within the associated white matter tracts remain less explored. To establish a causal relationship, such neural coordination must occur before the cognitive control response is generated. By studying the temporal dynamics of functional connectivity modulations between distinct cortical areas, we aim to gain a clearer understanding of the specific role of white matter neural coordination in verbal cognitive control.

Our objective was to construct a five-dimensional functional atlas that illustrates the neural substrates supporting verbal cognitive control. We refer to this technique as a ‘five-dimensional atlas’ because it emphasizes the *temporal dynamics* of task-related cortical modulations and *functional connectivity* through white matter tracts, all set within a *three-dimensional* space. This atlas effectively captures the dynamics of functional connectivity modulations within white matter tracts preferentially involved in word reading and the Stroop color naming task. The Stroop task, in which participants are asked to name the ink color of a word that represents a different color, is among the most common paradigms used to study verbal cognitive control (Stroop, 1992). This task is more demanding than word reading, as evidenced by prolonged response times across diverse populations, including healthy individuals and those with drug-resistant focal epilepsy (Homack and Riccio, 2004; Koga et al., 2011; Chen et al., 2022). Functional MRI (fMRI) and intracranial EEG (iEEG) studies underscore that the prefrontal and premotor cortices are more active during the Stroop task than word reading (Floden et al., 2011; Koga et al., 2011; Chmielewski and Beste, 2019). Other studies suggest that the posterior brain regions, including left fusiform, inferior temporal, and inferior parietal regions, are critically involved in word reading (Gaillard et al., 2006; Wu et al., 2011; Vidal et al., 2012; Kunii et al., 2013; Woolnough et al., 2022).

In the five-dimensional atlas presented in this study, functional connectivity refers to synchronous neural activity in different brain regions that are directly connected by white matter tracts (Kitazawa et al., 2023; Ono et al., 2023). A key methodological innovation is the use of direct streamlines between cortical regions as a defining criterion for functional connectivity, which enhances the biological plausibility of the observed connections. Previous fMRI studies have shown that when participants focused on word stimuli to be read, there was an increase in hemodynamic responses in posterior brain regions (Egner and Hirsch, 2005; Polk et al., 2008). Conversely, when participants ignored word stimuli to attend to color naming, increased activation was observed in the prefrontal/premotor areas, with a decrease in posterior brain regions. In line with these findings, our previous iEEG study of five patients revealed that Stroop task-specific neural activation predominantly involved the left prefrontal and premotor areas, particularly 300 to 600 milliseconds before responses (Koga et al., 2011). Based on these observations, we hypothesize that task-specific functional connectivity modulations would similarly occur several hundred milliseconds before responses.

To quantify and visualize task-preferential modulations of functional connectivity, the current study measured event-related high-gamma activity at 70–110 Hz (Kitazawa et al., 2023; Ono et al., 2023). This activity serves as a surrogate marker for both neural activation and deactivation, with a temporal resolution on the order of tens of milliseconds and signal fidelity over 100 times superior to scalp EEG (Ball et al., 2008). High-gamma amplitudes have been shown to correlate closely with hemodynamic activation in fMRI (Nir et al., 2007; Kunii et al., 2013), neuronal firing in single neuron recordings (Nir et al., 2007; Ray et al., 2008; Leszczyński et al., 2020), and glucose metabolism in positron emission tomography (Nishida et al., 2008). Notably, among wideband iEEG activities, surgical resection of cortical sites displaying event-related augmentation of high-gamma activity was the most predictive of postoperative cognitive declines (Sonoda et al., 2022). To visualize the white matter tracts that connect distinct cortical regions activated simultaneously during a particular moment of a task, we employed diffusion-weighted imaging (DWI) tractography (Sonoda et al., 2021; Sakakura et al., 2023). As a result, the current study provided a five-dimensional functional atlas showing the neural substrates supporting verbal cognitive control, by presenting the temporal dynamics of task-related cortical modulations and functional connectivity through white matter tracts animated on a three-dimensional space.

## 2. Material and methods

### 2.1. Participants

Inclusion criteria for this study included: (i) patients with focal epilepsy who underwent extraoperative intracranial EEG (iEEG) recording in Children’s Hospital of Michigan, Detroit (Nakai et al., 2017); (ii) age of 10 years or older at the time of the study; (iii) both right- and left-handed individuals; (iv) completion of word reading and Stroop color naming tasks as mentioned below. Exclusion criteria were: (i) presence of diffuse brain malformations (e.g., perisylvian polymicrogyria and megalencephaly) known to alter the anatomical landmarks for the central and calcarine sulci (Kitazawa et al., 2023), (ii) history of prior epilepsy surgery. Accordingly, we studied a consecutive series of seven native English-speaking patients who met the eligibility criteria mentioned above (age range: 10 – 17 years; four females; Table 1). All patients were right-handed. The study received approval from the Institutional Review Board at Wayne State University. Written informed consent was obtained from the parents or guardians of all patients, and written assent was secured from patients aged 13 years or older.

### 2.2. Acquisition and processing of iEEG and imaging data

For extraoperative iEEG recording, platinum grid electrodes with a 10 mm intercontact distance were surgically implanted. The electrode plates were stitched to adjacent plates and/or the edge of the dura mater to minimize movement post-placement. Subsequent extraoperative video-iEEG was continuously recorded for 3 – 5 days using a 192-channel Nihon Kohden Neurofax 1100A Digital System (Nihon Kohden America Inc, Foothill Ranch, CA). The recording was conducted at a rate of 1000 Hz with a band-pass ranging from 0.016 – 300 Hz. For subsequent analysis, we used a common average reference technique, averaging iEEG voltage across all electrode channels. We excluded channels affected by artifact, seizure onset zones (Asano et al., 2009), interictal spike discharges (Kural et al., 2020) or MRI lesions (Sakakura et al., 2022). Thus, a total of 547 nonepileptic subdural electrode sites were available for analysis.

**Fig. 1.**
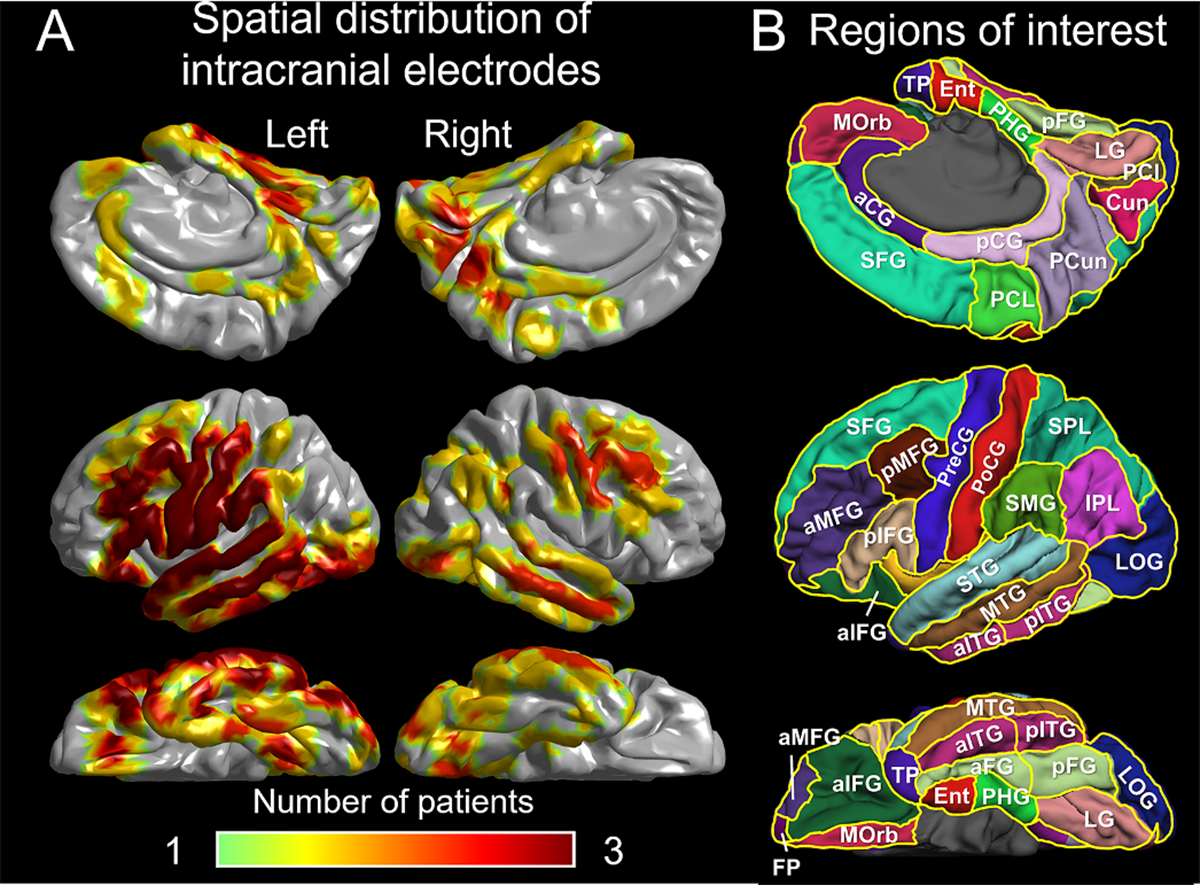
Spatial extent of non-epileptic electrode sites on a standard brain surface image. (A) This figure indicates the number of patients available at a given analysis mesh. (B) This figure indicates the locations of region of interests (ROIs) on the left hemisphere. aCG: anterior cingulate gyrus. aFG: anterior fusiform gyrus. aIFG: anterior inferior-frontal gyrus. aITG: anterior inferior-temporal gyrus. aMFG: anterior middle-frontal gyrus. Cun: cuneus. Ent: entorhinal gyrus. FP: frontal pole. IPL: inferior parietal lobule. LG: lingual gyrus. LOG: lateral occipital gyrus. MOrb: medial orbitofrontal gyrus. MTG: middle-temporal gyrus. pCG: posterior cingulate gyrus. PCl: pericalcarine cortex. PCL: paracentral lobule. PCun: precuneus. pFG: posterior fusiform gyrus. PHG: parahippocampal gyrus. pIFG: posterior inferior-frontal gyrus. pITG: posterior inferior-temporal gyrus. pMFG: posterior middle-frontal gyrus. PoCG: postcentral gyrus. PreCG: precentral gyrus. SFG: superior-frontal gyrus. SMG: supramarginal gyrus. SPL: superior parietal lobule. STG: superior-temporal gyrus. TP: temporal pole. Group-level regions of interest (ROIs) were employed only in those with at least five nonepileptic electrode sites. The number of non-epileptic electrode sites in each ROI is provided in Table S1.

We generated a three-dimensional (3D) surface image of the cerebral cortex using a preoperative 3-Tesla, T1-weighted spoiled gradient-recalled echo sequence MRI. By incorporating a computed tomography image taken immediately after the subdural electrode placement, we projected these electrodes onto the pial surface of the cerebral cortex, as demonstrated by Stolk et al. (2018). For a group-level analysis of iEEG high-gamma amplitude modulations during tasks, we standardized each electrode location to the FreeSurfer standard coordinates, commonly referred to as FSaverage (http://surfer.nmr.mgh.harvard.edu). Using the Desikan anatomical parcellation, we segmented each hemisphere of the cerebral cortex into 29 regions of interest (ROIs) (i.e., 58 ROIs in both hemispheres; Fig. 1; Desikan, et al., 2006). Our iEEG analysis was conducted at 28 of these ROIs, each containing at least 5 electrode sites (16 in the left and 12 in the right hemisphere; see Table S1).

### 2.3. Tasks

**Fig. 2.**
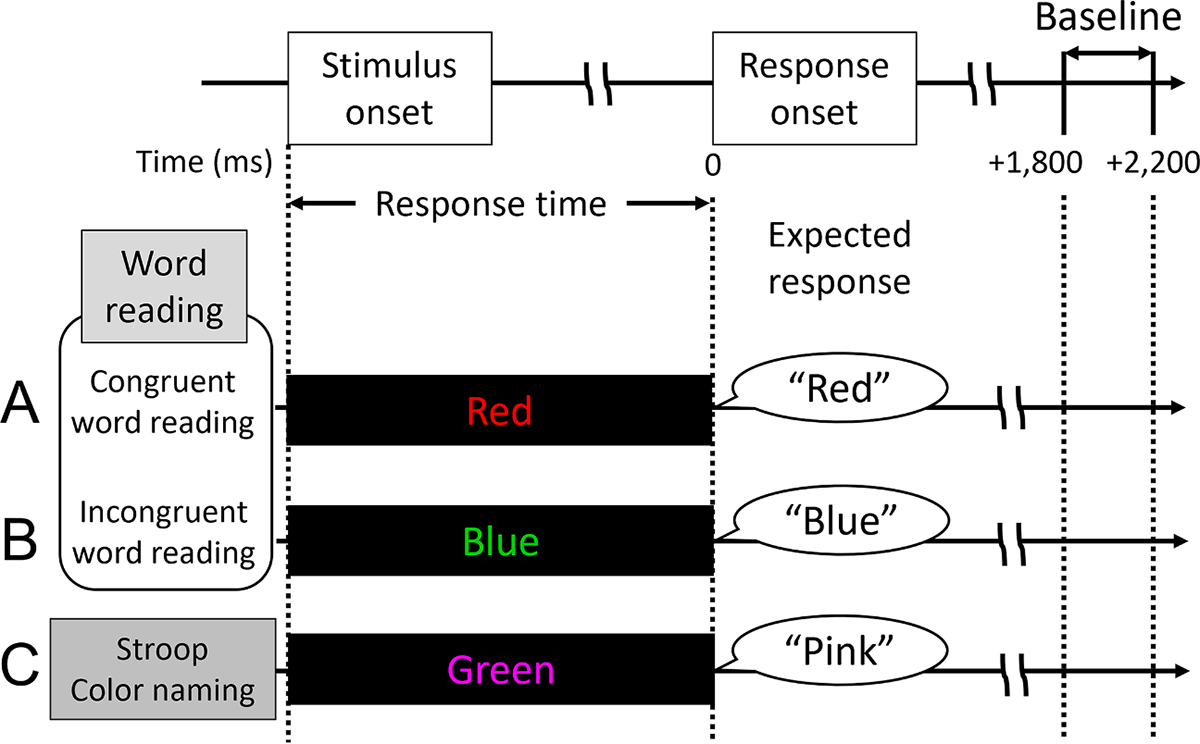
Tasks. (A) Congruent word reading task. (B) Incongruent word reading task. (C) Stroop color naming task. The response time is characterized as the period between the onset of the stimulus and the onset of the response. The baseline period for intracranial EEG analysis pertains to the 400-ms quiet period between 1800 and 2200 ms post-response onset.

All patients performed the following tasks 1–2 days after the placement of subdural electrodes. No patient experienced a seizure within 2 h prior to performing the tasks. Each task consisted of 40 trials. In these trials, patients were visually presented with one of four words (‘Red’, ‘Blue’, ‘Green’, or ‘Pink’) in a pseudorandom sequence (Koga et al., 2011). They were then instructed to provide a verbal response based on the following specific directions for each task:

- Congruent word reading task: In this task, each word was displayed in a matching ink color (e.g., ‘Red’ printed in red ink) (Fig. 2A). Patients were instructed to read the word aloud, ignoring the ink color (e.g., saying “red” when ‘Red’ in red ink was presented).
- Incongruent word reading task: Here, each word was displayed in a non-matching ink color (e.g., ‘Red’ in blue ink) (Fig. 2B). Again, patients were instructed to read the word aloud, disregarding the ink color (e.g., saying “red” when ‘Red’ in blue ink was displayed).
- Stroop color naming task: In this task, each word appeared in a non-matching ink color (Fig. 2C). This time, patients were instructed to name the ink color rather than the word (e.g., saying “blue” when ‘Red’ in blue ink was presented).

The visual stimulus was presented on a 17-inch LCD computer monitor positioned 60 cm in front of the patients. Stimuli were displayed at the center of the monitor against a black background for 5000 ms. The inter-stimulus interval varied randomly between 2000 and 2500 ms. Presentation Software (Neurobehavioral Systems Inc, Albany, CA) was used for stimulus delivery. Response time was defined as the period between stimulus onset and response onset. The exact timings of the stimulus onset and offset, as well as the patients’ responses, were integrated into the iEEG acquisition system through the DC input.

### 2.4. Measurement of high-gamma amplitude modulations during a task

We quantified the high-gamma amplitude (proportional to the square root of power) at each electrode site during each task by employing a time-frequency analysis. Our objective was to identify specific time points and ROIs where high-gamma amplitude was either augmented or attenuated (Sakakura et al., 2022; Kitazawa et al., 2023; Ono et al., 2023). The core of our time-frequency analysis was the complex demodulation algorithm, implemented in the BESA EEG Analysis Software (BESA GmbH, Gräfelfing, Germany; Papp and Ktonas, 1977; Hoechstetter et al., 2004). This approach involved multiplying each iEEG time-voltage signal with a complex exponential followed by a low-pass filter in increments of 10 ms and 5 Hz. Notably, with its Gaussian shape, the complex modulation algorithm closely mirrored a Gabor transformation. Its temporal and frequency domains exhibited a full width at half maximum of 15.8 × 2 ms and 7.1 × 2 Hz, respectively. For assessment of high-gamma modulations at each electrode site, we measured the percentage change in amplitude compared to the baseline mean, which was derived between 1800 and 2200 ms post-response onset; this baseline period was set effectively between response onset and stimulus onset. The analysis time window of interest encompassed (i) an 800 ms interval from 200 ms before to 600 ms after stimulus onset and (ii) an 800 ms interval from 600 ms before to 200 ms after the response onset.

### 2.5. Visualization of task-related modulations of high-gamma amplitude

**Fig. 3.**
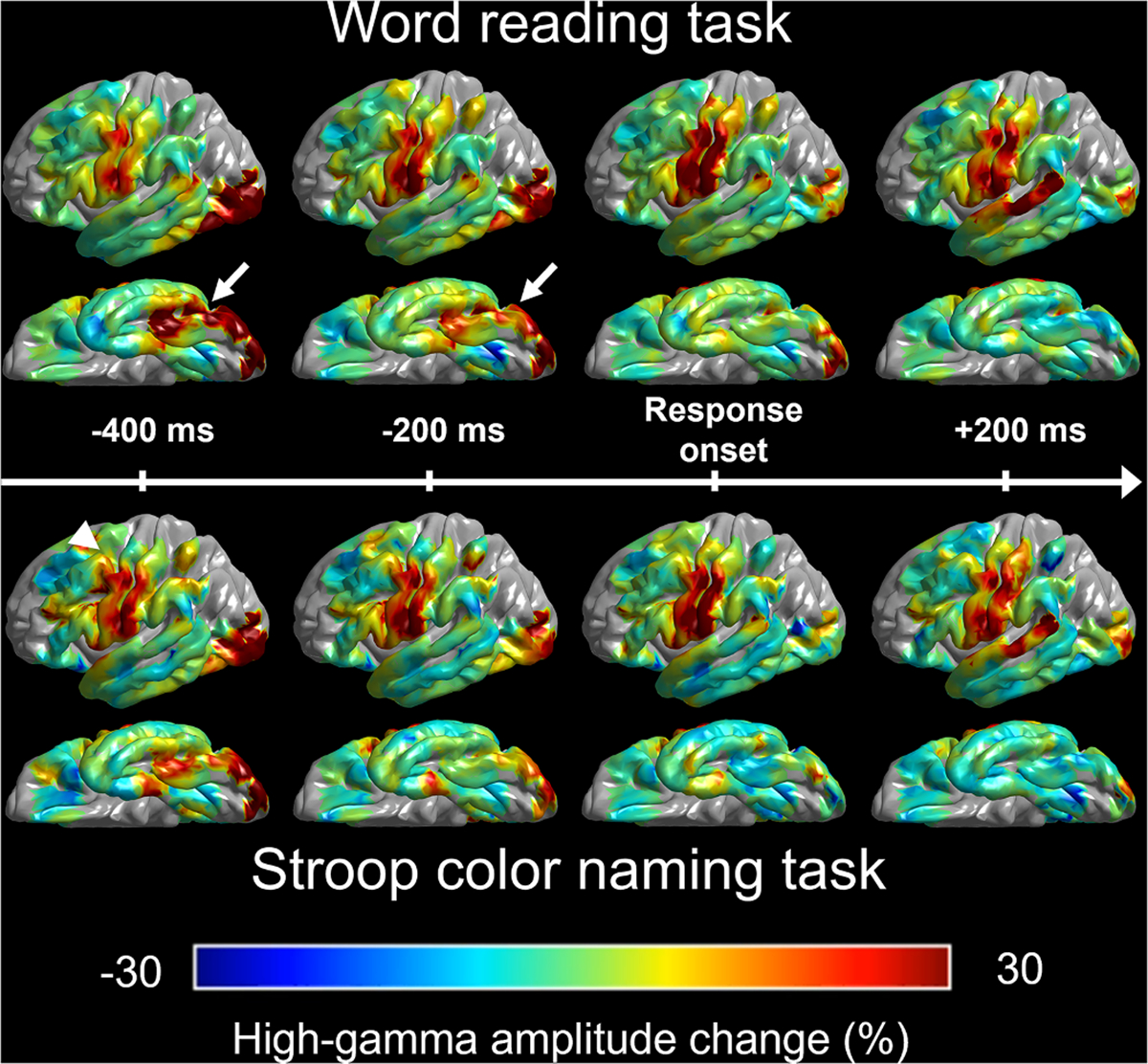
Task-related high-gamma amplitude modulations. The snapshots showcase the percent change of high-gamma amplitudes in comparison to the baseline mean. Upper: high-gamma modulations during word reading (i.e., average during congruent and incongruent word-reading tasks). Arrows indicate high-gamma augmentation in the posterior fusiform region. Lower: Stroop color naming. An arrowhead indicates high-gamma augmentation in the posterior middle frontal gyrus. For a comprehensive overview of the high-gamma amplitude changes during each task, please refer to Video S1.

Utilizing iEEG data from all 547 electrode sites, we animated the task-related high-gamma amplitude modulations every 10 ms throughout the analysis time windows. These modulations are presented as percentage changes relative to the baseline mean. For visualization, we projected these changes onto specified electrode sites on the spatially normalized FreeSurfer pial surface, “FSaverage,” and used interpolation within a 10 mm radius from the electrode center (Fig. 3; Video S1).

### 2.6. Region of interest (ROI) analysis of task-related high-gamma amplitude

**Fig. 4.**
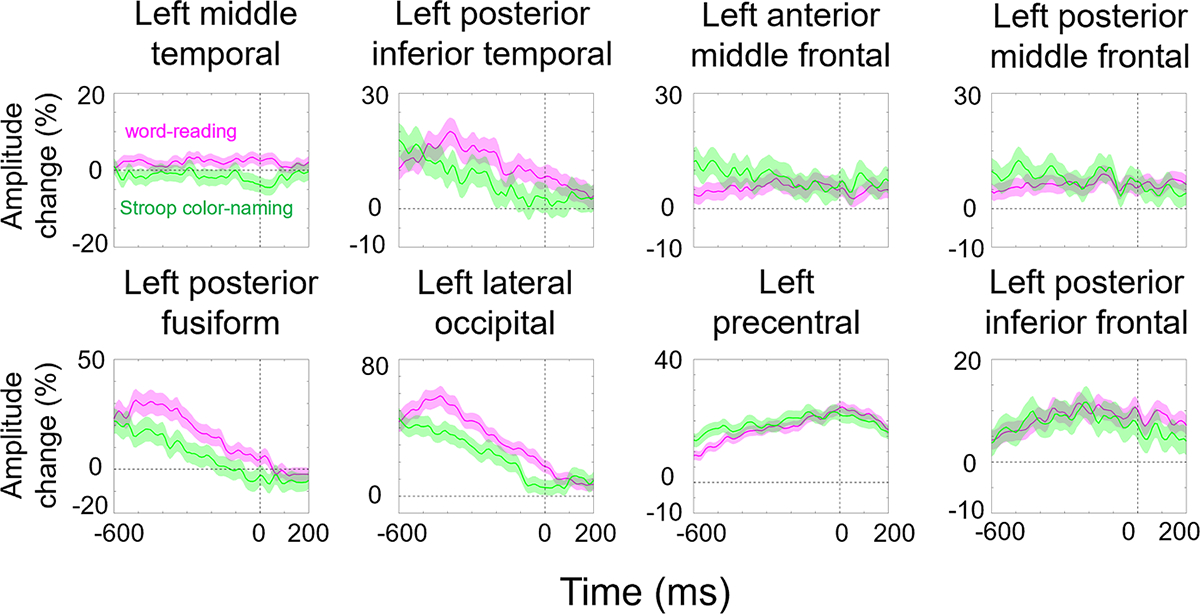
Task-related modulations of high-gamma amplitude. A given plot presents the percent change in high-gamma amplitudes at each anatomical ROI compared to the baseline mean at 1800 to 2200 ms after response onset. Magenta: word-reading (i.e., average of congruent and incongruent word reading tasks). Green: Stroop color-naming. Shade: 95 % confidence interval.

Within a specific ROI, we assessed whether the task-related high-gamma amplitude increased or decreased significantly compared to the baseline mean (Fig. 4). To test the null hypothesis – that the average task-related high-gamma modulations (expressed as % changes in amplitude) in each 10-ms time bin matched the baseline mean – we used a permutation test with 1000 permutations. We set a two-sided significance threshold of 5 %. Subsequently, we applied a false discovery rate (FDR) correction across an analysis time window of 800 ms (i.e., 80 time bins). If the permutation test indicated that the amplitude was different from the baseline mean for a duration of at least 40 ms (equivalent to three high-gamma oscillatory cycles), we considered the high-gamma amplitude as significantly modulated. The permutation test likewise determined when high-gamma amplitude differed between the word reading and Stroop color naming tasks.

### 2.7. Mixed model analysis to determine the independent effects of tasks on high-gamma amplitude

**Fig. 5.**
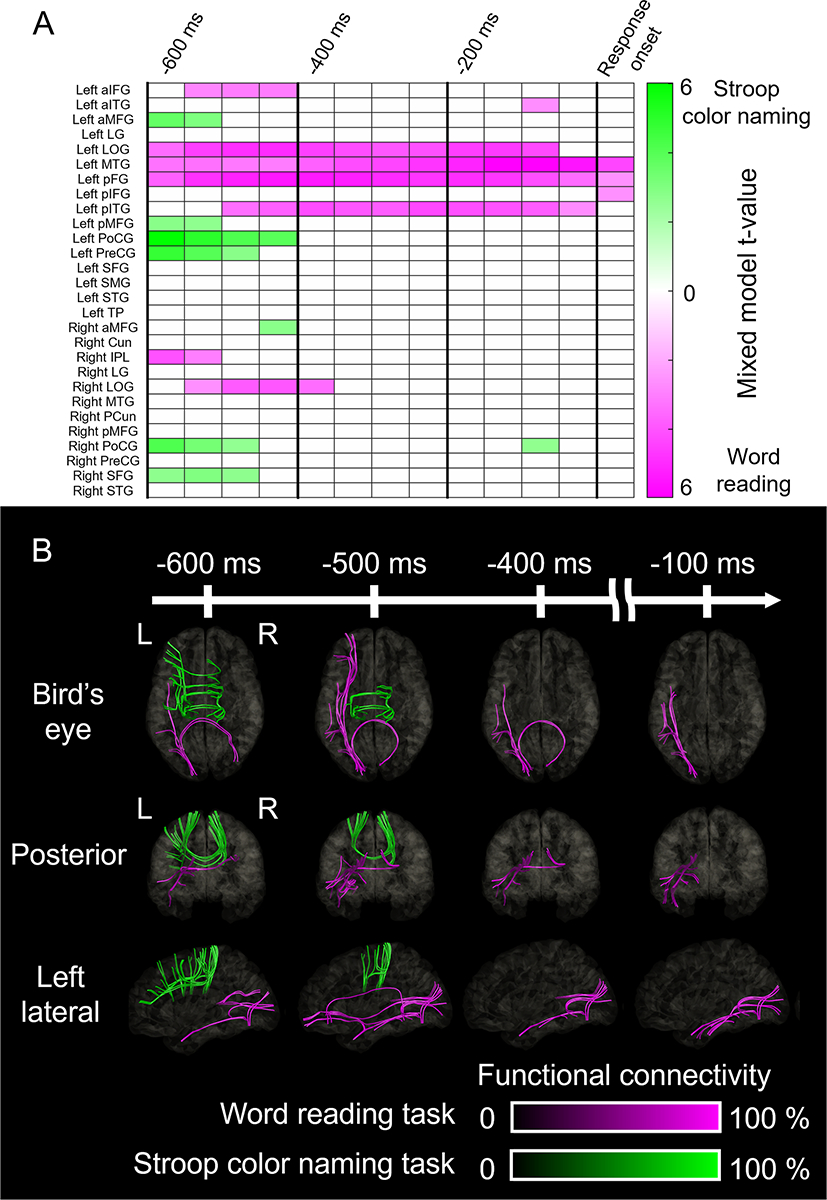
Cortical and white matter substrates preferentially supporting given tasks. (A) Spatiotemporal characteristics of high-gamma amplitudes differentially modulated during Stroop color naming versus word reading. Cells present mixed model t-values for specific regions of interest (ROIs) and their corresponding 200-ms time windows (e.g., −600 ms: a time window between −600 and −400 ms pre-response onset). These colors indicate when and where high-gamma amplitude was significantly higher (green) or lower (magenta) during Stroop color naming relative to word reading. Readers will find that high-gamma amplitude at the left posterior middle-frontal gyrus (pMFG) was higher during Stroop task compared to word reading at two time windows starting at −600 ms and −550 ms pre-response (i.e., between −600 and −400 ms and −550 and −350 ms before verbal responses). (B) Color-coded streamlines depict the spatiotemporal dynamics of functional connectivity enhancement between ROIs preferential to Stroop color naming (green) and word reading (magenta). For a comprehensive overview of the task-preferential network dynamics, please refer to Video S2. aIFG: anterior inferior-frontal gyrus. aITG: anterior inferior-temporal gyrus. aMFG: anterior middle-frontal gyrus. Cun: cuneus. IPL: inferior parietal lobule. LG: lingual gyrus. LOG: lateral occipital gyrus. MTG: middle-temporal gyrus. PCun: precuneus. pFG: posterior fusiform gyrus. pIFG: posterior inferior-frontal gyrus. pITG: posterior inferior-temporal gyrus. PoCG: postcentral gyrus. PreCG: precentral gyrus. SFG: superior-frontal gyrus. SMG: supramarginal gyrus. STG: superior-temporal gyrus. TP: temporal pole.

We aimed to pinpoint both ‘when’ and ‘in which ROI’ high-gamma amplitude differed between tasks (Fig. 5) to clarify the cortical substrate supporting verbal cognitive control. In this analysis, we controlled for potential confounding effects from clinical and epilepsy-related variables. Within each ROI, we employed a mixed model analysis technique (Sonoda et al., 2021; Sakakura et al., 2022) to ascertain if the high-gamma amplitude, taken in a 200-ms sliding window with a step of 50 ms, showed any differences between the word reading and the Stroop color naming. The fixed effect predictors encompassed: (i) task type (a value of 1 was assigned for the Stroop color naming), (ii) trial number (ranging from 1 to 40), (iii) age at surgery (quantified in years), (iv) sex (where female was designated as 1), (v) presence of an MRI-visible structural lesion (marked as yes with a value of 1), and (vi) the number of antiseizure medications taken immediately prior to the iEEG recording. Given the known correlation between a higher count of antiseizure medications and a more severe cognitive burden related to seizures (Kwan and Brodie, 2001), we incorporated the medication count as a predictor. For our analysis, both the patient and the intercept were categorized as random factor variables. If a difference in high-gamma amplitude between the tasks maintained an FDR-corrected, two-sided p-value of <0.05, we considered the difference to be significant independently of the effects of clinical and epilepsy-related variables. This correction took into account 364 comparisons conducted across 28 ROIs for 13 200-ms windows.

For interested readers, we employed another mixed model analysis to determine whether the high-gamma amplitude, measured in a 200-ms sliding window with a 50 ms step, showed any differences between the congruent and incongruent word reading tasks (Figure S1).

### 2.8. Visualization of task-preferential modulations of functional connectivity

To visualize the spatiotemporal characteristics of task-preferential functional connectivity modulations via white matter tracts, we created a dynamic tractography video using a method similar to those reported previously (Videos S2–S3; Kitazawa et al., 2023; Ono et al., 2023; Sakakura et al., 2023). The mixed model analysis, performed in the previous section, determined the 200-ms time windows in which high-gamma amplitude significantly differed between word reading and Stoop naming tasks at given pairs of cortical ROIs (Fig. 1B). We thereby declared the Stroop color naming task preferentially enhanced functional connectivity between white matter-connected ROIs if: [i] high-gamma amplitude was significantly and simultaneously higher during Stoop naming than word reading in both ROIs and [ii] these ROIs were connected by direct DWI streamlines. In turn, using the timing of high-gamma amplitude during word reading higher than Stroop color naming, we determined the spatiotemporal profiles of word reading-preferential enhancement of functional connectivity.

To visualize the anatomical white matter streamlines, we utilized open-source DWI data from 1065 healthy participants (Yeh et al., 2018; http://brain.labsolver.org/diffusion-mri-templates/hcp-842-hcp-1021). This approach has been previously validated in our work (Sonoda et al., 2021), as discussed in detail below. To identify white matter tracts underlying task-preferential functional connectivity modulations, we placed seeds at cortical ROIs revealing significant and simultaneous positive (or negative) task effects on high-gamma amplitude, as determined by the mixed model analysis. The DSI Studio script (http://dsi-studio.labsolver.org/) visualized tractography streamlines directly connecting these cortical ROIs within the Montreal Neurological Institute standard space. The fiber tracking parameters used were a quantitative anisotropy threshold of 0.05, a maximum turning angle of 70° and a streamline length of 20 to 250 mm. A board-certified neurosurgeon (K.S.) excluded streamlines involving the brainstem, basal ganglia, thalamus, or cerebrospinal fluid space from the tractography analysis. The resulting dynamic tractography videos - sliding every 50 ms during the period between 600 ms before response onset and 200 ms after response onset - visualized the spatiotemporal dynamics of white matter streamlines linking cortical ROIs with significant task effects on high-gamma amplitude. Fig. 5 and Video S2 illustrate the dynamics of functional connectivity modulations favoring the Stroop color naming when contrasted with the word reading.

For interested readers, we have provided Figure S1 and Video S3, which clarify the dynamics of functional connectivity modulations favoring incongruent word reading compared to congruent word reading.

## 3. Results

### 3.1. Behavioral observations

The Stroop effect was confirmed by the difference in response times between tasks. The mean response time was 826 ms (standard deviation [SD]: 172 ms) during the congruent word reading, 1034 ms (SD: 190 ms) during the incongruent word reading, and 1211 ms (SD: 234 ms) during the Stroop color-naming. The mixed model analysis, incorporating the Stroop task as fixed effect predictor and patient and intercept as random factors, revealed that the response time during Stroop color naming was longer than during word reading (t-value: 14.8; mixed model coefficient: 280 ms; 95 % confidence interval [95 %CI]: 243 to 317; uncorrected p-value: 2.08 × 10^−44^; degrees of freedom [DF]: 838). Another mixed model analysis revealed that the response time during incongruent word reading was longer than congruent word reading (t-value: 12.2; mixed model coefficient: 226 ms; 95 %CI: 189 to 262; uncorrected p-value: 1.88 × 10^−30^; DF: 558).

### 3.2. Task-related high-gamma amplitude modulations

Fig. 3 and Video S1 present the spatiotemporal dynamics of task-related high-gamma modulations in comparison to the baseline mean. Visual assessment indicates that high-gamma augmentation commonly involved the lateral occipital areas right after stimulus onset, the left precentral region maximally at response onset, and the superior temporal gyrus (STG) afterward. Below, we have provided the statistical results indicating the spatiotemporal profiles of task-preferential high-gamma augmentation.

Fig. 4 presents task-related high-gamma modulations in each ROI. The permutation test revealed that high-gamma amplitude during word reading (i.e., average of congruent and incongruent word reading tasks) was larger than that during Stroop color naming in the left occipitotemporal areas during the pre-response period. The maximum difference in high-gamma amplitude between tasks was 22.2 % in the lateral occipital area at 430 ms pre-response onset (uncorrected p-value: <0.0001), 17.4 % in the posterior fusiform gyrus at 370 ms pre-response onset (uncorrected p-value: <0.0001), 10.6 % in the posterior inferior temporal gyrus (pITG) at 400 ms pre-response onset (uncorrected p-value: <0.0001), and 7.6 % in the middle temporal gyrus (MTG) at 40 ms post-response onset (uncorrected p-value: <0.0001).

The permutation test revealed that high-gamma amplitude during Stroop color naming was larger than that during word reading in the left frontal lobe areas during the pre-response period. The maximum difference in high-gamma amplitude between tasks was 9.0 % in the anterior middle frontal gyrus (aMFG) at 590 ms pre-response onset (uncorrected p-value: <0.0001), 6.9 % in the posterior middle frontal gyrus (pMFG) at 490 ms pre-response onset (uncorrected p-value: 0.0020), and 6.1 % in the precentral gyrus at 570 ms pre-response onset (uncorrected p-value: <0.0001).

### 3.3. Independent effects of tasks on high-gamma modulations

The mixed model analysis revealed that, independently of the effects of clinical and epilepsy-related variables, high-gamma amplitude during word reading was larger than that during Stroop color naming in the left occipitotemporal areas during the pre-response period. The maximum mixed model effect was 16.6 % (95 %CI: 9.9 % to 23.4 %; uncorrected p-value: 1.5 × 10^−6^) in the lateral occipital gyrus during the 200-ms period between −500 and −300 ms pre-response onset, 14.0 % (95 %CI: 8.7 % to 19.3 %; uncorrected p-value: 2.9 × 10^−7^) in the posterior fusiform gyrus between −500 and −300 ms pre-response, 7.7 % (95 %CI: 4.1 % to 11.3 %; uncorrected p-value: 3.1 × 10^−5^) in the pITG between −400 and −200 ms pre-response, and 4.6 % (95 %CI: 2.8 % to 6.3 %; uncorrected p-value: 3.4 × 10^−7^) in the MTG between −200 ms pre-response and response onset.

Conversely, high-gamma amplitude during Stroop color naming was larger than during word reading in the left frontal lobe areas during the pre-response period. The maximum mixed model effect was 5.9 % (95 % CI: 2.7 % to 9.0 %; uncorrected p-value: 2.6 × 10^−4^) in the aMFG, 4.0 % (95 %CI: 1.2 % to 6.8 %; uncorrected p-value: 5.3 × 10^−3^) in the pMFG, 4.3 % (95 %CI: 2.5 % to 6.1 %; uncorrected p-value: 2.0 × 10^−6^) in the precentral gyrus. The maximum effect in these three ROIs was noted at a 200-ms period between −600 and −400 ms pre-response onset.

An ancillary mixed model analysis indicated that left postcentral and precentral high-gamma amplitude was larger during incongruent than congruent word reading. The maximum mixed model effect was 6.5 % (95 %CI: 4.3 % to 8.6 %; uncorrected p-value: 7.7 × 10^−9^) in the left postcentral region and 3.7 % (95 %CI: 1.7 % to 5.8 %; uncorrected p-value: 4.1 × 10^−4^) in the left precentral region. The maximum effect in these three ROIs was noted at a 200-ms period between −550 and −350 ms pre-response onset.

### 3.4. Task-preferential modulations of functional connectivity

Fig. 5 and Video S2 present the spatiotemporal dynamics of task-preferential modulations of functional connectivity through white matter pathways. As detailed in the Supplementary Document, the mutual dependence among fixed effect variables did not account for the word reading- or Stroop color naming-preferential functional connectivity enhancement described below.

Word reading-preferential enhancement of functional connectivity, as contrasted with Stroop color naming, took place during the pre-response period. By −600 ms pre-response onset, word reading-preferential functional connectivity enhancement involved [a] the posterior callosal fibers between the left and right lateral occipital regions, [b] the left vertical occipital fasciculus between the lateral occipital and posterior fusiform regions, and [c] the left inferior longitudinal fasciculus between the posterior fusiform and middle temporal regions. By −500 ms pre-response onset, word reading-preferential functional connectivity enhancement involved [d] the left inferior fronto-occipital fasciculus between the lateral occipital and aIFG, [e] the left uncinate fasciculus between the middle temporal and aIFG, and [f] the left arcuate fasciculus between pITG and aIFG. Word reading-preferential functional connectivity enhancement in [b] the left vertical occipital fasciculus and [c] the left inferior longitudinal fasciculus was sustained until response onset.

Stroop color naming-preferential enhancement of functional connectivity, as contrasted with word reading, also took place during the pre-response period. Between −600 and −300 ms pre-response onset, Stroop color naming-preferential functional connectivity enhancement involved [a] the anterior callosal fibers between the left and right pre-central gyri as well as the left pMFG and right SFG, and [b] the left frontal u-fibers between the pMFG, aMFG, and precentral gyri.

An ancillary analysis to assess the effect of word-ink color incongruency within reading tasks indicated that the incongruent word reading-preferential enhancement of functional connectivity involved (a) the callosal fibers between the left and right postcentral gyri between −500 and −300 ms pre-response, and (b) the superior longitudinal fasciculus between the right postcentral gyrus and the right aMFG between −450 and −200 ms pre-response (Figure S1; Video S3).

## 4. Discussion

### 4.1. Summary

The five-dimensional functional atlas we employed integrates spatial, temporal, and connectivity data, allowing us to analyze not only where these pathways are located but also how they interact over time during verbal tasks. By capturing dynamic changes in functional connectivity and accurately mapping the white matter tracts, this atlas provides a comprehensive framework for understanding verbal cognitive control. In this study, we specifically explored the network dynamics underlying verbal cognitive control by comparing them to those supporting automatic reading. We characterized the spatiotemporal patterns of functional connectivity enhancement within and between brain hemispheres. Notably, the Stroop color naming task was associated with more intense enhancement of functional connectivity in the left frontal u-fibers and anterior callosal fibers, extending from the left middle-frontal and the primary sensorimotor areas. These task-specific enhancements in functional connectivity commonly occurred in a period between 350 and 600 ms before the patients responded. These observations have improved our understanding of the brain’s ability to execute complex verbal tasks by prioritizing relevant linguistic information from our surroundings and inhibiting automatic responses. In contrast, word reading was associated with more intense enhancement of functional connectivity in the white matter pathways originating from the left occipital-temporal region. This includes the posterior callosal fibers, as well as the left vertical occipital, inferior longitudinal, inferior fronto-occipital, and arcuate fasciculi. These observations effectively help us better understand the brain network processes in word reading.

### 4.2. Stroop task-preferential functional connectivity enhancement within the left hemisphere

In the present study, the Stroop color naming task, as compared to word reading, induced more enhanced functional connectivity between the left MFG and sensorimotor cortices through the frontal u-fibers in a period between 350 and 600 ms prior to response onset. This observation is consistent with fMRI observations that the left MFG was among the cortical areas showing hemodynamic activation enhanced during Stroop color naming as compared to word reading (Adleman et al., 2002; Floden et al., 2011; Chmielewski and Beste, 2019). Prior studies reported that cortico-subcortical lesions, such as stroke or hemorrhage, involving the left MFG were causally associated with impaired performance in Stroop color naming (Stuss et al., 2001; Alexander et al., 2007). Studies of patients with multiple sclerosis reported that impaired performance in Stroop color naming was associated with demyelinating white matter lesions in the left frontal subcortical area (Foong et al., 1999), white matter microstructural abnormalities in the left frontal-parietal network (Ratzan et al., 2023), and reduction of resting-state functional connectivity in the left frontal-parietal network (Manca et al., 2019). An intracranial study reported that direct electrical stimulation of the left pMFG sites showing Stroop-specific high-gamma augmentation 300–600 ms prior to verbal responses elicited transient naming impairment (Koga et al., 2011). Another intracranial study reported that direct electrical stimulation of the left pMFG, compared to that of the left pIFG, more frequently impaired selection of required language when switch was required (either from L1 to L2 or vice versa) (Sierpowska et al., 2018). Studies of healthy participants reported that excitation of the left dorsolateral prefrontal region (proximal to aMFG) using 10-Hz repetitive transcranial magnetic stimulation (TMS) reduced response times in both Stroop color naming and word reading but did not modify the Stroop interference; the response time remained longer during Stroop color naming than reading (Vanderhasselt et al., 2006; Li et al., 2017; Parris et al., 2021). In contrast, direct electrical stimulation of the left pre-central gyrus frequently induces speech arrest (Breshears et al., 2015; Nakai et al., 2017; Lu et al., 2021). Taken together, we consider that the Stroop-preferential connectivity enhancement through the left frontal u-fibers between the left MFG and sensorimotor cortices would reflect dynamic network processing for attentive selection of required verbal actions (i.e., color naming in this study) in response to color word stimuli.

### 4.3. Stroop task-preferential functional connectivity enhancement between hemispheres

We found that the Stroop color naming task, as compared to word reading, induced more enhanced functional connectivity through the anterior callosal fibers in a period between 300 and 600 ms prior to response onset and that these fibers connected between the left MFG/sensorimotor cortices and the right SFG/sensorimotor cortices. This observation is consistent with fMRI observations that the right SFG was among the cortical areas showing hemodynamic activation enhanced during Stroop color naming as compared to word reading (Adleman et al., 2002; Milham et al., 2003; Melcher and Gruber, 2009). A study of 91 healthy adults reported that increased cortical thickness of the right SFG was predictive of better performance in Stroop color naming (Lavagnino et al., 2016). In contrast, cortico-subcortical lesions involving the right SFG were causally associated with impaired performance in Stroop color naming (Vendrell et al., 1995; Alexander et al., 2007) and No-Go tasks requiring inhibitory responses (Verfaellie and Heilman, 1987; Pickton et al., 2007). A study of patients with multiple sclerosis reported that demyelinating lesions involving the right SFG area were associated with slow responses in Stroop color naming task (Pujol et al., 2001). An intraoperative study of patients with brain tumor in the right frontal lobe reported that electrical stimulation of the white matter adjacent to the anterior corpus callosum impaired performance in Stroop color naming (Puglisi et al., 2019). Studies using TMS reported that stimulation over the right sensorimotor cortex transiently suppressed voluntary tonic hand movements of the right hand in individuals with the anterior corpus callosum on MRI but not in those without (Meyer et al., 1995; Di Lazzaro et al., 1999). An intracranial case study reported that direct electrical stimulation of the anterior corpus callosum in the right hemisphere induced transient mutism (Völk et al., 2019). In another intracranial case study using a Go/No-Go task, the right SFG showed high-gamma augmentation approximately 300 ms prior to stop response (Swann et al., 2012). Taken together, we consider that the Stroop-preferential connectivity enhancement through the anterior callosal fibers would reflect dynamic network processing for suppression of automatic responses (i.e., word reading in this study) in response to color word stimuli.

### 4.4. Word reading-preferential functional connectivity enhancement within the left hemisphere

As compared to the Stroop task, word reading induced more enhanced functional connectivity between the left lateral occipital and posterior fusiform regions through the vertical occipital fasciculus and between the left posterior fusiform and middle temporal regions through the inferior longitudinal fasciculus by 600 ms prior to response. This observation is consistent with fMRI observations that the left posterior fusiform (McCandliss et al., 2003; Devlin et al., 2006) and middle temporal regions (Choi et al., 2014; Schuster et al., 2020) were among the cortical areas showing hemodynamic activation enhanced during word reading tasks. Prior studies reported that lesions involving the left posterior fusiform (Hillis et al., 2005; Gaillard et al., 2006; Roberts et al., 2015) or middle temporal region (Turken and Dronkers, 2011; Li et al., 2021) resulted in impaired reading skills. Direct electrical stimulation of the left fusiform region was reported to induce pure alexia (Mani et al., 2008; Sabsevitz et al., 2020), whereas that of the middle temporal sites is suggested to frequently induce anomia in addition to alexia (Zemmoura et al., 2015). Intracranial studies have shown that, during word reading tasks, high-gamma augmentation in the left posterior fusiform region precedes augmentation in the left middle temporal regions, whereas such high-gamma augmentation sustained in both regions at least 250–500 ms after stimulus onset (Vidal et al., 2012; Kaestner et al., 2021; Liu et al., 2021; Woolnough et al., 2022). Taken together, we consider that word reading-preferential connectivity enhancement between the left lateral occipital and posterior fusiform regions would reflect processing for identifying the letter and word forms, whereas that between the left posterior fusiform and middle temporal regions would reflect processing for connection of word and its meaning.

### 4.5. Word reading-preferential functional connectivity enhancement between hemispheres

As compared to the Stroop task, word reading induced more enhanced functional connectivity between the left and right lateral occipital regions through the posterior callosal fibers by 550 ms prior to response. This observation is consistent with the fMRI observations that the left and right lateral occipital regions were among the cortical areas showing hemodynamic activation enhanced during word reading tasks (Vigneau et al., 2005; Neudorf et al., 2022). Prior studies have reported that callosotomy, or lesions involving the posterior callosal fibers, resulted in alexia, particularly for word stimuli presented in the left-sided visual field (Silver et al., 1988; Vaddiparti et al., 2021). Intracranial study has demonstrated that, during a word reading task, both left and right lateral occipital regions show high-gamma augmentation with 100 ms after stimulus onset (Wu et al., 2011). Another study using stereoencephalography demonstrated that electrical stimulation of homotopic cortical areas elicits responses that propagate through the corpus callosum within 50 ms (Mitsuhashi et al., 2021). Taken together, we consider that the enhancement of word-reading preferential connectivity via the posterior callosal fibers reflects the process of integrating word stimuli processed in the visual cortex of each hemisphere.

### 4.6. Novelty

Our movie atlas uniquely depicts the millisecond-scale dynamics of functional connectivity changes through direct white matter tracts. Our animation movie illuminates the differences between verbal cognitive control and automatic word reading. We are not aware of any study that visualizes the rapid dynamics of functional connectivity through specific white matter pathways during tasks involving these cognitive processes. The visualization is based on high-gamma modulations recorded intracranially from non-epileptic sites during specific tasks. One task emphasized verbal cognitive control over word reading, while the other prioritized word reading. To estimate the enhancement of functional connectivity through white matter tracts, we embraced the principle that cortical regions displaying simultaneous high-frequency neural responses are likely involved in coordinated interactions (Buonomano and Merzenich, 1998; Singer, 2018). Our previous iEEG study revealed that distant cortical areas demonstrating concurrent task-related high--gamma augmentation are frequently connected by direct white matter pathways exhibiting increased effective connectivity as defined by early neural responses to single-pulse electrical stimulation (Sonoda et al., 2021). Expanding upon these findings, our recent iEEG studies have produced video atlases showcasing the rapid dynamics of functional connectivity modulations within white matter during various activities, including naming (Kitazawa et al., 2023) and eye opening and closure (Ono et al., 2023); in these studies, we defined functional connectivity modulations based on significant and simultaneous high-gamma augmentation, exceeding chance levels.

### 4.7. Methodological considerations

Due to our small sample size, our analysis was confined to 28 ROIs (16 in the left and 12 in the right hemisphere). This limitation is inherent in iEEG studies, as electrode placement is determined solely by clinical necessity, not research objectives. As such, some ROIs might have had less than ideal sample sizes. Technical limitations in tractography and a limited number of electrode sites per ROI restricted our ability to reduce the size of these ROIs further. Despite these methodological limitations, our observations are generally consistent with prior literature as discussed above, and none of our reported findings fundamentally contradict generally accepted knowledge in this field. Thus, we believe our study provides unique spatiotemporal information useful for constructing a biologically plausible model to explain the cortical and white matter substrates supporting verbal cognitive control and word reading processes. Looking ahead, we expect more stereo-electroencephalography data from deep cortical and thalamic regions. Future improvements in tractography sensitivity and larger collaborative studies may allow for more detailed connectivity analysis across smaller cortical and subcortical ROIs with robust statistical power.

We used task-related high-gamma amplitude to determine the timing of neural activation at specific ROIs and to characterize the dynamics of functional connectivity across ROIs for the following reasons. Our prior iEEG study of 65 patients revealed that resection of sites showing task-related amplitude augmentation of high-gamma activity, among many other frequency bands, best predicted postoperative cognitive outcomes (Sonoda et al., 2022). It is feasible to expect the temporal dynamics of task-related high-gamma amplitude modulations in given ROIs to be grossly similar across individuals (Nakai et al., 2017; Ikegaya et al., 2019). However, it is infeasible to expect the phase of high-gamma activity in ROIs to be shared by different individuals. Task-related high--gamma augmentation is characterized by amplitude augmentation of broadband activity not confined to a specific narrow frequency band (Lachaux et al., 2012). Thus, we did not analyze coherence or traveling waves dependent on the phase of iEEG waveforms at given ROIs. Instead, we assessed functional connectivity using a method agnostic to the phase of iEEG activity.

Our aim was to characterize iEEG high gamma-based functional connectivity modulations across major white matter pathways typical in the general population. It is important to note that our study was not designed to identify age-related changes in DWI measures or to pinpoint abnormal DWI streamlines in patients with focal epilepsy. Our mixed model analysis lacked sufficient statistical power to assess the impact of age on task-related high-gamma amplitudes. A much larger sample size would be required for such purposes. Investigators need a sample size of 100 or more to identify significant white matter development during adolescence, as indicated in previous DWI studies (Asato et al., 2010; Baum et al., 2022). In our previous study (Sonoda et al., 2021), we combined iEEG data from the nonepileptic cortices of 37 children with drug-resistant focal epilepsy (aged 5–20) with DWI data from healthy individuals. We utilized both individual and open-source DWI data for this analysis. Our findings showed a strong correlation (Pearson’s *r* = 0.8) between propagation velocities computed using both data sources, supporting the utility of normative DWI data from the Human Connectome Project.

## Supplementary Material

1

2

3

4

## Figures and Tables

**Table 1:** Patient profile.

Patient	Age in years	Sex	Sampled hemisphere	Presence of MRI-visible lesions	Number of antiseizure medications
1	14	Female	Left	No	3
2	10	Female	Left	Yes	2
3	17	Male	Left	No	2
4	17	Male	Left	Yes	1
5	17	Female	Left	No	1
6	17	Female	Right	No	2
7	14	Male	Right	Yes	3

## Data Availability

The iEEG data are available at https://openneuro.org/datasets/ds004859/versions/1.0.0 (doi:https://doi.org/10.18112/openneuro.ds004859.v1.0.0).

## Abbreviations:

CI: confidence interval
EEG: electroencephalography
ERP: event-related potential
FDR: false discovery rate
fMRI: functional magnetic resonance imaging
iEEG: intracranial electroencephalography
ROI: regions of interest
