# Supplementary material for: Dynamic functional connectivity in verbal cognitive control and word reading
Source: Neuroimage. Author manuscript; Available in PMC 2024 Oct 24. (PMC11500755; doi:10.1016/j.neuroimage.2024.120863)
Supplement: 4 [file NIHMS2028430-supplement-4.docx]

**Supplementary document in**

**Dynamic functional connectivity in verbal cognitive control and word reading**

Kazuki Sakakura ; Matthew Brennan ; Masaki Sonoda ;

Takumi Mitsuhashi ; Aimee F Luat ; Neena I Marupudi ; Sandeep Sood ; Eishi Asano

This supplementary document includes

**Table S1;**

**Figure S1;**

**Video legends S1-S3;**

**Ancillary analysis examining the mutual dependence among fixed effect variables.**

| **Region of interests** | **Left** | **Right** |
| --- | --- | --- |
| aCG: anterior cingulate gyrus | 3 (1) | 0 (0) |
| aFG: anterior fusiform gyrus | 4 (2) | 1 (1) |
| aIFG: anterior inferior-frontal gyrus | 19 (4) | 0 (0) |
| aITG: anterior inferior-temporal gyrus | 11 (3) | 3 (2) |
| aMFG: anterior middle-frontal gyrus | 27 (4) | 7 (2) |
| Cun: cuneus | 2 (1) | 6 (2) |
| Ent: entorhinal gyrus | 2 (1) | 1 (1) |
| FP: frontal pole | 0 (0) | 0 (0) |
| IPL: inferior parietal lobule | 4 (3) | 10 (2) |
| LG: lingual gyrus | 7 (3) | 9 (2) |
| LOG: lateral occipital gyrus | 17 (4) | 9 (2) |
| MOrb: medial orbitofrontal gyrus | 3 (2) | 0 (0) |
| MTG: middle-temporal gyrus | 36 (5) | 22 (2) |
| pCG: posterior cingulate gyrus | 3 (1) | 4 (2) |
| PCl: pericalcarine cortex | 0 (0) | 1 (1) |
| PCL: paracentral lobule | 0 (0) | 2 (1) |
| PCun: precuneus | 3 (1) | 11 (3) |
| pFG: posterior fusiform gyrus | 8 (4) | 4 (2) |
| PHG: parahippocampal gyrus | 2 (2) | 3 (1) |
| pIFG: posterior inferior-frontal gyrus (BA 44 and 45) | 31 (5) | 2 (1) |
| pITG: posterior inferior-temporal gyrus | 13 (4) | 3 (2) |
| pMFG: posterior middle-frontal gyrus | 20 (5) | 12 (2) |
| PoCG: postcentral gyrus | 40 (5) | 8 (2) |
| PreCG: precentral gyrus | 49 (5) | 9 (2) |
| SFG: superior-frontal gyrus | 9 (3) | 6 (3) |
| SMG: supramarginal gyrus | 32 (5) | 2 (2) |
| SPL: superior parietal lobule | 3 (1) | 3 (1) |
| STG: superior-temporal gyrus | 44 (5) | 10 (2) |
| TP: temporal pole | 5 (2) | 2 (1) |
| **Total** | 397 (5) | 150 (3) |

**Table S1. The number of artifact-free, nonepileptic electrode sites used for region of interest (ROI)-based analysis.** A total of 28 ROIs (16 in the left and 12 in the right hemisphere) contained at least 5 electrode sites and were included in the group-level statistical analysis.


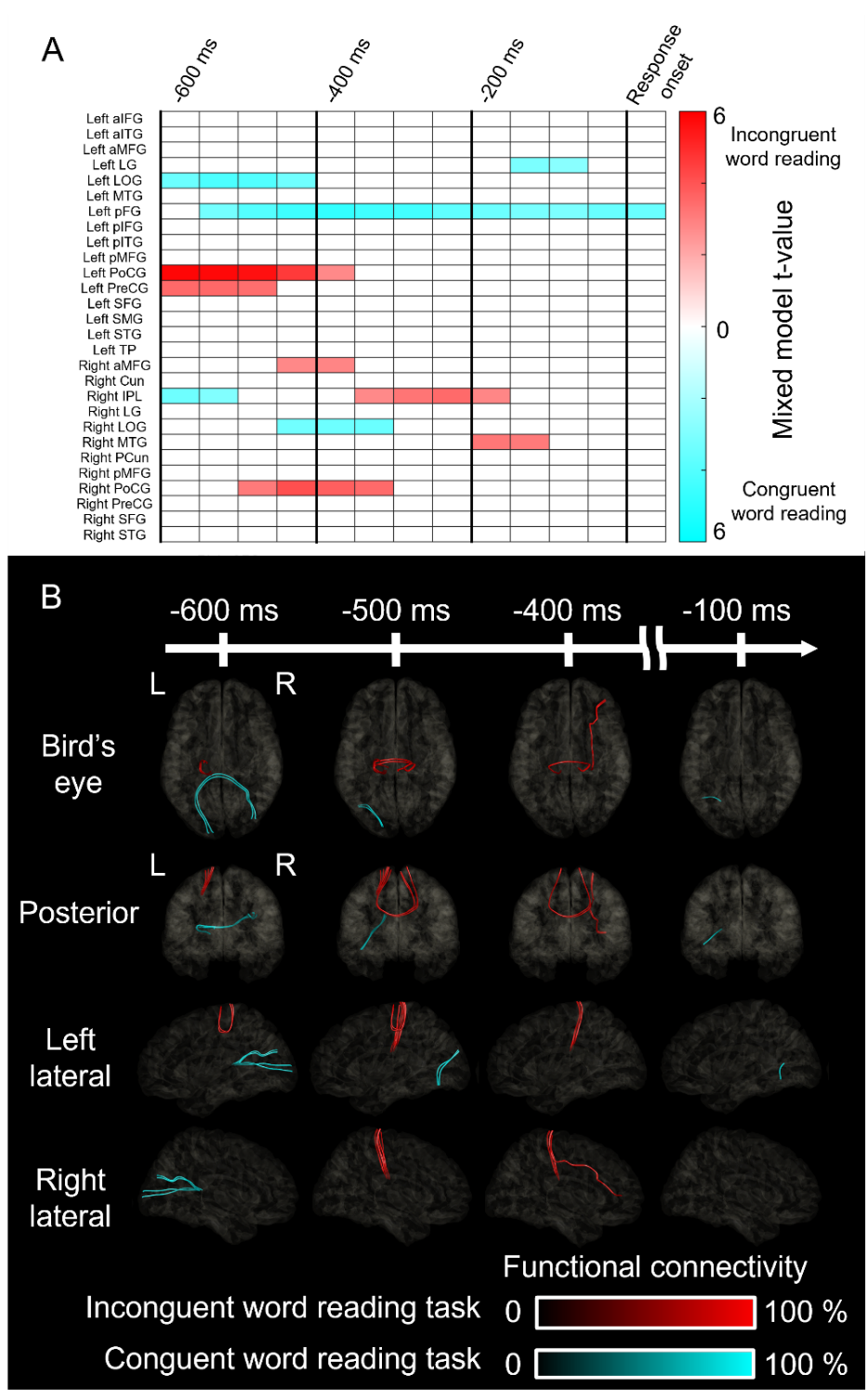


**Figure S1. Cortical and white matter substrates preferentially supporting given reading tasks.** (A) Spatiotemporal characteristics of high-gamma amplitudes differentially modulated during incongruent versus congruent word reading. Cells present mixed model t-values for specific regions of interest (ROIs) and their corresponding 200-ms time windows (e.g., -600 ms: a time window between -600 and -400 ms pre-response onset). These colors indicate when and where high-gamma amplitude was significantly higher (red) or lower (cyan) during incongruent word reading relative to congruent word reading. (B) Color-coded streamlines depict the spatiotemporal dynamics of functional connectivity enhancement between ROIs preferential to incongruent word reading (red) and congruent word reading (cyan). For a comprehensive overview of the task-preferential network dynamics, please refer to **Video S3**.

**Video legends**

**Video S1. High-gamma amplitude modulations during tasks.** The animation video showcases the percent change of high-gamma amplitudes in comparison to the baseline mean.

00:00 – 00:14: Left: Stroop color naming.

Right: Word reading (i.e., average of congruent and incongruent ones).

00:14 – 00:26: Left: Incongruent word reading.

Right: Congruent word reading.

**Video S2. Cortical and white matter substrates preferentially supporting given tasks.** Color-coded streamlines depict the spatiotemporal dynamics of functional connectivity enhancement between ROIs preferential to Stroop color naming (green) and word reading (magenta). 0 ms: a 200-ms time window between response onset and 200 ms post-response onset.

**Video S3. Cortical and white matter substrates preferentially supporting given reading tasks.** Color-coded streamlines depict the spatiotemporal dynamics of functional connectivity enhancement between ROIs preferential to incongruent word reading (red) and congruent word reading (cyan). 0 ms: a 200-ms time window between response onset and 200 ms post-response onset.

**Ancillary analysis examining the mutual dependence among fixed effect variables.**

In the cortical regions of interest (ROIs) associated with significant task-preferential functional connectivity modulations (as shown in **Figure 5**), we assessed whether such task-preferential modulations could be attributed to the mutual dependence among fixed effect variables. To this end, we computed the Variance Inflation Factor (VIF) for the fixed effect variables (age, sex, MRI lesion, and number of antiseizure medications). In the left hemisphere, none of these variables exhibited an excessive VIF (e.g., >5), indicating no substantial mutual dependence affecting our functional connectivity findings.

In the right hemisphere, VIFs exceeding 5 were noted in several ROI which showed significant task-preferential modulations of functional connectivity at some time windows. These ROIs included the inferior parietal lobule (IPL), lateral occipital gyrus (LOG), postcentral gyrus (PoCG), and superior frontal gyrus (SFG). Consequently, we repeated the mixed model analysis in these ROIs, including only the task type and trial number as fixed effect variables.

The ancillary mixed model analysis replicated significant word reading-preferential high-gamma augmentation in the right IPL at -550 ms pre-response onset (t-value: -3.00; uncorrected p-value: 0.003), in the right LOG at -550 ms (t-value: -2.61; uncorrected p-value: 0.009), -500 ms (t-value: -3.84; uncorrected p-value:1.3 x 10^-4^), -450 ms (t-value: -3.93; uncorrected p-value: 9.2 x 10^-5^), and -400 ms pre-response onset (t-value: -3.43; uncorrected p-value: 0.001). Thus, the word reading-preferential functional connectivity enhancement found in the right hemisphere cannot be merely attributed to the unwanted effect of mutual dependence among fixed effect variables.

The mixed model analysis, incorporating only the task type and trial number as fixed effect variables, likewise replicated significant Stroop color naming-preferential high-gamma augmentation in the right PoCG at -600 ms (t-value: 4.15; uncorrected p-value: 3.6 x 10^-5^), -550 ms (t-value: 3.25; uncorrected p-value: 0.001), and -500 ms pre-response onset (t-value: 2.59; uncorrected p-value: 0.01), and also in the right SFG at -600 ms (t-value: 2.68; uncorrected p-value: 0.008), -550 ms (t-value: 2.99; uncorrected p-value: 0.003), and -500 ms pre-response onset (t-value: 2.67; uncorrected p-value: 0.008). Thus, the Stroop color naming-preferential functional connectivity enhancement in the right hemisphere cannot be merely attributed to the unwanted effect of mutual dependence among fixed effect variables.
